# Subcapsular Pancreatic Pseudocyst of the Right Hepatic Lobe: A Rare Case Report and Literature Review

**DOI:** 10.3390/diseases14050174

**Published:** 2026-05-15

**Authors:** Nutu Vlad, Laurentiu Budaca, Alexandra Ciubotariu, Florina-Delia Andriesi-Rusu, Mircea Florin Costache, Gigel Sandu, Andrei Cristea, Cătălin Sfarti

**Affiliations:** 1 ”Saint Spiridon” Hospital Iasi, 700111 Iasi, Romania; nutu.vlad@umfiasi.ro (N.V.); blaurentiu33@gmail.com (L.B.); alexandra.ciubotariu99@gmail.com (A.C.); costache.mircea@gmail.com (M.F.C.); gigelsandu@gmail.com (G.S.); andrei_epi@yahoo.com (A.C.); cvsfarti@gmail.com (C.S.); 2 Faculty of Medicine, “Grigore T. Popa” University of Medicine and Pharmacy Iasi, 700115 Iasi, Romania

**Keywords:** extrapancreatic pseudocyst, intrahepatic pancreatic pseudocyst, subcapsular hepatic pancreatic pseudocyst, giant extrapancreatic pseudocyst, laparoscopic treatment of extrapancreatic large pseudocyst, case report

## Abstract

The pancreatic pseudocyst is a collection of pancreatic fluid surrounded by a non-epithelialized wall comprising granulation tissue and fibrosis, occurring in approximately 10% of patients diagnosed with acute pancreatitis and in 20–38% of those with chronic pancreatitis. Most pseudocysts are situated in the pancreatic head and pancreatic body, but about 20% develop in extrapancreatic locations. We present the case of a 46-year-old male patient diagnosed with chronic alcohol pancreatitis with acute exacerbation, who developed a large pancreatic pseudocyst with subcapsular location in the right hepatic lobe; this was successfully treated by laparoscopic surgical drainage, with no postoperative complications and no recurrence of the pseudocyst. The computed tomography scan and postoperative biochemical analysis of the intracystic fluid played a key role in establishing the diagnosis of this rare condition. An intrahepatic pancreatic pseudocyst is a rare location for pancreatic pseudocysts, but one located in the right hepatic lobe is extremely rare. The treatment of intrahepatic pancreatic pseudocysts may be conservative, though endoscopic, percutaneous, or surgical drainage may be necessary. The presence of symptoms, signs of extrinsic compression, or complications require drainage of the pseudocyst. The “take-away” lesson learned from this case: surgical treatment for pancreatic pseudocysts located subcapsularly in the liver may be considered when they are very large, or when minimally invasive treatment has not been effective.

## 1. Introduction

Pseudocysts are the most common pancreatic cystic lesions. They represent a collection of pancreatic fluid surrounded by a non-epithelialized wall comprising granulation tissue and fibrosis [1,2,3]. Pseudocysts occur most frequently as a complication of chronic pancreatitis and less commonly as a complication of acute pancreatitis [1,4,5]. An acute pseudocyst presents as a collection of pancreatic fluid bordered by early granulation tissue, typically developing within 3–4 weeks following an episode of acute pancreatitis [1,2,6]. This collection may resolve spontaneously in approximately 50% of cases, although spontaneous regression is less likely in the case of large pseudocysts exceeding 6 cm [1]. Conversely, pseudocysts that increase in size on serial evaluations and become symptomatic require surgical treatment [1]. Most pseudocysts are situated in the cephalic and body portion of the pancreas, but about 20% grow in extrapancreatic locations, with unusual sites including the kidney, liver, pleura, or even the mediastinum [1,3]. The first case of an intrahepatic pancreatic pseudocyst was published in 1974 [7].

We hereby present the case of a patient with acute-on-chronic pancreatitis who developed a giant pancreatic pseudocyst in an exceptionally rare location: subcapsular in the right hepatic lobe. The pseudocyst was successfully treated with laparoscopic surgical drainage, with no postoperative complications and no recurrence.

## 2. Case Report

We present a case of a 46-year-old male patient, a chronic alcohol consumer, with medical history in the Gastroenterology Clinic for multiple episodes of toxic acute pancreatitis occurring within the same year; the patient presented to the emergency department with nausea, frequent vomiting at home, and intense diffuse abdominal pain radiating to the right scapula and the right lumbar region, with symptoms onset approximately 12 h prior to admission. The patient reported multiple previous hospitalizations, the most recent occurring about 3 weeks earlier in the Gastroenterology Clinic.

On physical examination, the patient appeared in moderately impaired general condition, afebrile, with a slightly distended, depressible abdomen with spontaneous pain and palpable tenderness, with pain radiating to the right shoulder and right lumbar region. Giordano’s sign was negative, and bowel transit was present for both gas and stool. Palpation of the right hypochondrium revealed a firm, poorly defined, immobile mass measuring approximately 8–10 cm, which became markedly painful upon deep palpation.

**Figure 1:**
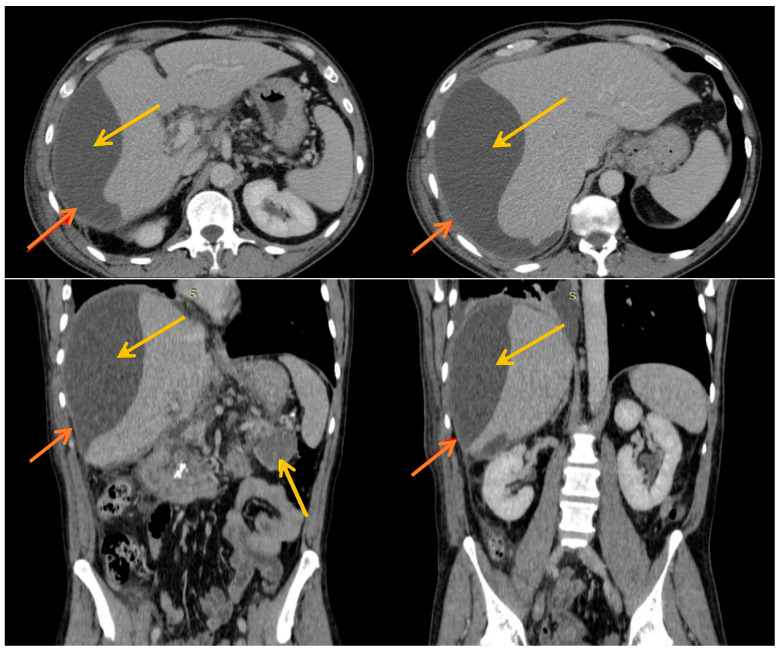
CT scan images: yellow arrow = pseudocyst; red arrow = hepatic capsule. The biconvex lens form specific for the hepatic subcapsular pseudocyst is observed.

Laboratory tests revealed anemia (hemoglobin = 10.5 g/dL), inflammatory markers (leukocytes = 14,680/mm^3^ with neutrophils = 11,240/mm^3^, C-reactive protein = 14.83 mg/dL), elevated serum lipase = 727 U/L and serum amylase = 415 U/L, and cholestasis, with unfavorable progression during the first days of hospitalization, as evidenced by rising alkaline phosphatase (ALP) and gamma-glutamyl transferase (GGT) levels (ALP = 59 → 224 U/L, GGT = 56 → 267 U/L). The patient was monitored with serial ultrasound examinations in the Gastroenterology Clinic. After 3 to 4 days following admission, a partially defined 14 cm collection was identified overlying the right hepatic lobe. Consequently, an urgent contrast-enhanced computed tomography (CT) scan was performed. Compared to the CT scan obtained during the previous hospitalization (14 days earlier), a new fluid density formation was observed, exerting compressive effects on the right hepatic lobe, with an inverted multilocular contour, subcapsular location, and dimensions of 177 × 80 × 190 mm. Considering the sudden onset and rapid progression, a subcapsular hepatic pancreatic pseudocyst was suspected. Additionally, a previously described pseudocyst was noted at the pancreatic body, measuring 15 × 24 × 24 mm, with no change in size (Figure 1).

**Figure 2:**
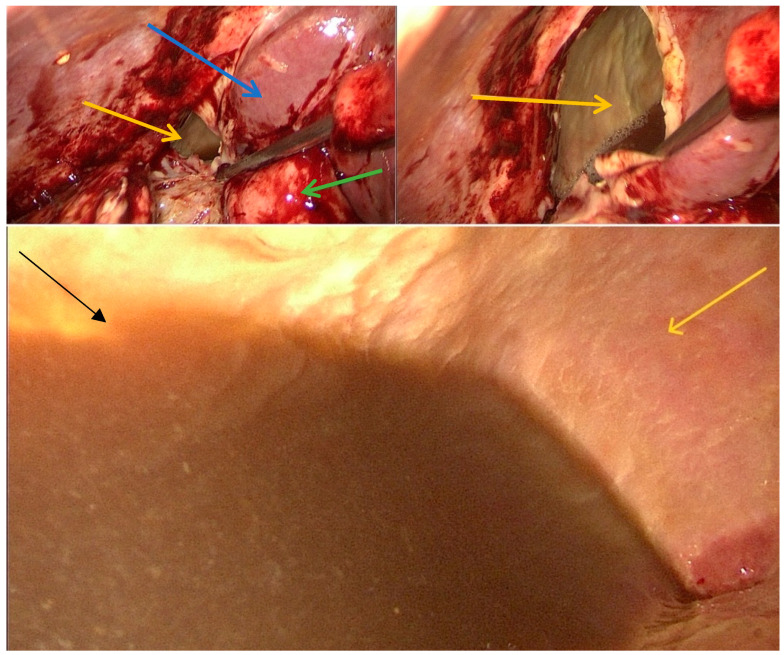
Intraoperative images: yellow arrow = pseudocyst, intracystic appearance; blue arrow = right hepatic lobe; green arrow = gallbladder; black arrow = intracystic fluid.

Given the patient’s severe pain and the compressive effect on the hepatic pedicle with progression of cholestasis syndrome, the patient was transferred to the surgical department where we decided to do a laparoscopic surgical drainage. Intraoperatively, a pronounced peritoneal inflammatory reaction was observed in the upper abdomen, predominantly in the right hypochondrium, with perihepatic adhesions. During visceral mobilization, a sizable lesion located in segments V–VI was identified; it ruptured upon suction-assisted manipulation and discharged a dark-colored fluid. Samples were collected for biochemical and microbiological analysis (results: CEA within normal limits, and microbiological analysis negative). Following aspiration, a giant residual cavity with walls showing granulation tissue and fibrosis was revealed (Figure 2).

A passive intracystic drainage tube was placed (in the remaining cavity of the pseudocyst), along with a second passive drain adjacent to the gallbladder and a third drain positioned in the Douglas pouch. Postoperative treatment was with analgesics, proton pump inhibitors, and anticoagulants for thromboembolism prophylaxis. Postoperative antibiotic therapy was not administered; only one prophylactic intraoperative dose of Cefuroxim 1.5 g single dose was given. No somatostatin analogs were required. The patient had a favorable postoperative course, with progressive reduction in drainage, resolution of inflammatory and cholestatic syndromes, and disappearance of symptoms. On the 10th postoperative day, the drain tubes were removed, and patient was discharged on postoperative day 11. Biochemical analysis of the intracystic fluid confirmed the diagnosis of a subcapsular hepatic pancreatic pseudocyst: amylase = 65,221 U/L and lipase >193,000 U/L. CEA dosage was within normal limits. Subsequent imaging examinations (multiple ultrasounds and MR cholangiography on postoperative day 7) demonstrated complete regression of the subcapsular hepatic pseudocyst. Postoperative MRCP did not reveal communication of the pseudocyst with a pancreatic duct. Imaging control (abdominal ultrasound) 1 month after discharge showed no recurrence of the pseudocyst.

Acknowledged limitations: single-patient experience; 1 month follow-up is too short to claim definitive non-recurrence (recommend 6- and 12-month imaging); and preoperative ductal anatomy was not delineated by MRCP.

## 3. Discussion

A pancreatic pseudocyst is a complication that occurs in up to 10% of patients diagnosed with acute pancreatitis [8]. It is a collection of pancreatic fluid surrounded by a non-epithelialized wall comprising granulation tissue and fibrosis [2,6]. A pancreatic pseudocyst requires between 4 and 6 weeks to form the well-defined wall [2]. Casado et al. suggested that the development time of an intrahepatic pancreatic pseudocyst may be much shorter than that of an intra-abdominal pancreatic pseudocyst [2]. The lack of data in the literature cannot support this observational data.

Most pseudocysts are located in the cephalic and body region of the pancreas, or in the peripancreatic tissues; however, atypical locations have also been reported, including intrahepatic, perisplenic, perirenal, retroperitoneal, pleural, and even mediastinal sites [8,9].

Intrahepatic pancreatic pseudocysts are in an extremely rare location, with few cases described in the literature [2]. Most of them are located in the left hepatic lobe and much less frequently in the right hepatic lobe. These can be single or with multiple hepatic locations [2]. The maximum number of multiple intrahepatic pseudocysts reported in a single patient, to our knowledge, is eight [9].

Between 1970 and 2016, only 54 cases of intrahepatic pancreatic pseudocysts were published in the literature [10]. Demeusy et al. observed that most cases were described in men, and the average size of the pseudocysts was 9.5 cm diameter, most of which were in the left hepatic lobe [10]. Guesmi et al. also observed that intrahepatic pancreatic pseudocysts are more frequent in men than women, with a sex ratio 3:1 [5].

The pathophysiological mechanism involves the proteolytic effect of pancreatic enzymes, which dissect anatomical planes [1,8]. Two mechanisms have been proposed for the intrahepatic extension of a pancreatic pseudocyst [7,11]. The first involves the accumulation of pancreatic fluid in the prerenal space, which subsequently erodes the posterior parietal peritoneum into the omental bursa [2,9,11]. From the omental bursa, pancreatic fluid ascends along the gastrohepatic ligament to the liver, resulting in a subcapsular pseudocyst in the left hepatic lobe [3,11]. This explains the affinity for localization in the left hepatic lobe. The second mechanism involves the dispersion of pancreatic fluid along the hepatoduodenal ligament, leading to, as in our patient, a subcapsular hepatic pseudocyst in the right lobe, or even with penetration of the porta hepatis, resulting in intraparenchymal pseudocysts [1,2,9,11]. The precise mechanism of intrahepatic pancreatic pseudocyst development is not fully understood. Small pseudocysts often resolve spontaneously, whereas larger ones may have a highly variable course over time, with periods ranging from 6 days to 2 months [2,7]. These collections have distinct imaging characteristics on CT, as subcapsular pseudocysts are biconvex in shape, while intraparenchymal pseudocysts are located away from the hepatic capsule, adjacent to the branches of the porta hepatis [3,10,11].

In our case, the preoperative differential diagnosis was guided by the rapid development of this large collection (177 × 80 × 190 mm) over 2 weeks (documented on serial CT scans and ultrasound exams), in the context of a recent acute pancreatitis episode approximately 2–3 weeks prior, as well as by the imaging characteristics of the lesion. The choice of treatment depends on cyst location, size, patient impact, and the presence of communication with the main pancreatic duct [3,10]. The communication of the pseudocyst with the main pancreatic duct can be demonstrated by MRCP, which delineates the anatomy of the main pancreatic duct and biliary tree [3]. Treatment options include main pancreatic duct stenting, endoscopic drainage, and occasionally surgical management [3,4,7,12,13]. The decision for surgical intervention in this case was made due to severe pain, the progressive cholestatic syndrome, and the large volume of the pseudocyst. A definitive diagnosis was established after biochemical analysis of the intracystic fluid, which revealed markedly elevated amylase and lipase levels, excluding the possibility of malignancy or infection [3,14].

The differential diagnosis of an intrahepatic pancreatic pseudocyst should be made with simple cysts, hydatid cysts, liver abscesses, or other liver formations [3,7].

Due to the rarity of such cases, there is no treatment guideline for pancreatic pseudocysts with subcapsular hepatic location [8,11]. Therefore, clinicians will judge the case and determine treatment based on the patient’s symptoms, the location of the pseudocyst, its size, and its associated comorbidities [10]. Considering that some pseudocysts may regress spontaneously, imaging monitoring and conservative treatment may be one of the possible therapeutic options [11]. In up to 50% of cases, pancreatic pseudocysts may disappear spontaneously [5]. In the absence of a response to conservative treatment, percutaneous drainage of the pseudocyst, endoscopic drainage, or surgical treatment remain the last treatment options available [8,11]. The percutaneous or surgical drainage is usually well tolerated [10]. However, to date, there are no clear criteria that require cyst drainage [1,5]. There is no data in the literature regarding the recurrence of extrapancreatic pseudocysts after endoscopic, percutaneous, or surgical drainage.

In the absence of treatment, pseudocysts can become complicated by bleeding, infection, fistula, compression, or rupture of the pseudocyst [8,10,13]. One of the causes of infection in pseudocysts can be following endoscopic retrograde cholangiopancreatography [13].

Up to 10% of cases of chronic pancreatitis associate pancreatic pseudocysts with arterial pseudoaneurysms [11]. Intracystic bleeding can cause severe pain in patients who associate pancreatic pseudocyst with pseudoaneurysms [11].

Pseudocysts with perirenal localization may be complicated by perirenal abscess, obstructive hydronephrosis, pseudoaneurysm, or renin-mediated hypertension [8].

Zhu et al. reported a case of an extremely rare complication in a patient with secondary Budd–Chiari syndrome caused by a giant intrahepatic pancreatic pseudocyst compressing the hepatic vein. In this case, the treatment was percutaneous drainage [7]. The choice of percutaneous drainage was aimed at obtaining slow drainage of the pseudocyst in order to prevent circulatory system disturbance caused by the sudden decrease in intra-abdominal hypertension [7]. Zhu et al. recommend percutaneous drainage when there are signs of huge intra-abdominal hypertension [7].

Another extremely rare complication was reported by Patidar et al., where an extremely rare vascular complication of an intrahepatic pancreatic pseudocyst ruptured into the inferior vena cava. In this case, the pancreatic pseudocyst was located to the caudate lobe [6].

The particularity of our case consists of the rapid development of a giant pancreatic pseudocyst (18 cm in diameter) with a rare extrapancreatic location—subcapsular right hepatic lobe. From the data we have from the published literature, this is one of the largest pancreatic pseudocysts located subcapsular right hepatic. Also, the case is fully explored, as we have a diagnosis of certainty, with specific history and symptoms; clear, complete imaging; typical appearance of CT images; intraoperative images edifying for diagnosis; and a diagnosis of certainty supported by extremely high values of amylase in the intracystic fluid.

The challenge was to support the diagnosis of extrapancreatic subcapsular hepatic pseudocyst and differentiate it from other diagnoses, such as acute peripancreatic fluid collection (APFC), acute necrotic collection (ANC), or walled-off necrosis (WON). In accordance with the revision of the Atlanta classification and definitions by international consensus for classification of acute pancreatitis, the APFC only applies to areas of peripancreatic fluid, without the feature of a pseudocyst and with no definable wall encapsulating the collection, as in our case report [15,16]. In our case, the collection had its own wall with fibrosis, and the content was only liquid, clear, without non-liquid densities, and without necrosis or intracystic bleeding.

A walled-off necrosis is a mature, encapsulated collection, heterogeneous with liquid and non-liquid density, with varying degrees of loculations [15,17,18,19,20]. Our collection was definitely not heterogeneous. It only had liquid densities with a fibrotic wall encapsulation.

Acute necrotic collection contains fluid and necrosis, with heterogeneous and non-liquid density, and no definable wall encapsulating the collection [15].

In our opinion, it is necessary to develop a diagnostic and treatment guideline for extrapancreatic pseudocysts.

Our experience is limited to issuing recommendations, but our results may be useful for centralization of data. We believe that for establishing the diagnosis, both the patient’s history and imaging studies are important, which mandates CT scans and MRCPs. For hospitals where imaging studies are limited or not performed, the diagnosis may be suspected following an abdominal ultrasound correlated with the patient’s history. In this case, the diagnosis of certainty will be established after determining the dosage of amylases and lipases from the pseudocyst fluid. Regarding treatment, small pseudocysts can be treated conservatively. Medium and large pseudocysts, as well as symptomatic ones, require drainage, either endoscopically or percutaneously, and when the first two cannot be performed or have not led to the pseudocyst’s remission, surgical treatment, either laparoscopic or classical, may be indicated.

An extensive electronic search of PubMed, from the 1970s to the present, with the keywords “intrahepatic pancreatic pseudocyst”, found 65 relevant results. From these, case reports similar to ours, or reports of limited case series, were selected. Guesmi et al. found 22 cases of pancreatic pseudocysts located intrahepatic or subcapsularly in the liver published between 1990 and 2009 [5]. The etiology identified in these cases was predominantly chronic pancreatitis, followed by alcohol and gallstones.

The symptoms identified in published case reports were either non-specific, absent, or abdominal pain associated with nausea or diarrhea. In a few cases, patients noted a palpable abdominal mass [5]. Of these, the most common symptom was abdominal pain.

Hepatic pancreatic pseudocysts can be single or multiple. Those located subcapsularly are mainly single, while those located intrahepatic are often multiple, located either in a single hepatic lobe or in both hepatic lobes.

Of the 22 cases analyzed by Guesmi et al., 15 cases benefited from percutaneous ultrasound or CT drainage, four from surgical treatment, and three without specific treatment. Death was recorded in three patients, while the others had a favorable evolution [5].

Demeusy et al. found 41 conclusive articles from 1970 to 2016, presenting 54 similar cases. In this systematic review, the mean size of hepatic pancreatic pseudocysts was 9.5 cm. The treatment chosen was also percutaneous drainage of the pseudocyst, surgical treatment, or endoscopic treatment, with ERCP (endoscopic retrograde pancreatography) and stenting of the pancreatic duct [10].

From our point of view, future research directions concerning treatment methods depend on the intrahepatic location (intraparenchymal versus subcapsular) and the size of the pseudocyst. It is necessary to establish clear criteria for when conservative treatment, percutaneous drainage, or surgical treatment is recommended.

## 4. Conclusions

Subcapsular hepatic pancreatic pseudocysts are an extremely rare complication of pancreatitis. Intrahepatic pseudocysts are often overlooked when evaluating hepatic lesions, but the appearance of a collection in the subcapsular hepatic region in a patient with known chronic pancreatitis or following an acute pancreatitis episode should be considered. Although the clinical presentation is non-specific, computed tomography and analysis of intracystic fluid play a key role in diagnosing this condition. Therapeutic management varies depending on case-specific factors, and the approach should be multidisciplinary.

## Data Availability

The original contributions presented in this study are included in the article. Further inquiries can be directed to the corresponding author.
